# Molecular Characterization by Whole-Genome Sequencing of Clinical and Environmental *Serratia marcescens* Strains Isolated during an Outbreak in a Neonatal Intensive Care Unit (NICU)

**DOI:** 10.3390/diagnostics12092180

**Published:** 2022-09-09

**Authors:** Alessandra Piccirilli, Sabrina Cherubini, Fabrizia Brisdelli, Paolo Fazii, Andrea Stanziale, Susanna Di Valerio, Valentina Chiavaroli, Luigi Principe, Mariagrazia Perilli

**Affiliations:** 1 Department of Biotechnological and Applied Clinical Sciences, University of L’Aquila, 67100 L’Aquila, Italy; 2 Clinical Microbiology and Virology Unit, Pescara Public Hospital, 65122 Pescara, Italy; 3 Neonatal Intensive Care Unit, Pescara Public Hospital, 65123 Pescara, Italy; 4 Liggins Institute, The University of Auckland, Auckland 1141, New Zealand; 5 Clinical Pathology and Microbiology Unit, “S. Giovanni di Dio” Hospital, 88900 Crotone, Italy

**Keywords:** WGS, *S. marcescens*, antibiotic resistance genes

## Abstract

The whole-genome sequencing (WGS) of eighteen *S. marcescens* clinical strains isolated from 18 newborns hospitalized in the Neonatal Intensive Care Unit (NICU) at Pescara Public Hospital, Italy, was compared with that of *S. marcescens* isolated from cradles surfaces in the same ward. The identical antibiotic resistance genes (ARGs) and virulence factors were found in both clinical and environmental *S. marcescens* strains. The *aac(6′)-Ic*, *tetA(41)*, *bla*_SRT-3_, *adeFGH*, *rsmA*, and PBP3 (D350N) genes were identified in all strains. The SRT-3 enzyme, which exhibited 10 amino acid substitutions with respect to SST-1, the constitutive AmpC β-lactamase in *S. marcescens*, was partially purified and tested against some β-lactams. It showed a good activity against cefazolin. Both clinical and environmental *S. marcescens* strains exhibited susceptibility to all antibiotics tested, with the exception of amoxicillin/clavulanate.

## 1. Introduction

*Serratia marcescens* is a Gram-negative bacterium that has been considered, for a long time, to be an environmental organism because of its ability to survive in surfaces, soil, and aquatic reservoirs [1]. Chromosomal-encoded genetic factors such as efflux pump systems, porins, virulence factors, and the capacity to acquire antibiotic resistance genes (ARGs) by horizontal transfer facilitate the survival of *S. marcescens* in different environments, causing nosocomial infections [2,3,4,5]. Additionally, *S. marcescens* has been found as bacterial contaminant of the blood donors, after venipuncture, or of blood platelet concentrates [6]. Over the last decades it has emerged as clinical pathogen in different hospital settings, in particular in intensive care units (ICUs) and neonatal intensive care units (NICUs) [7,8,9,10,11,12]. *S. marcescens* is responsible for a wide range of asymptomatic and severe clinical manifestations such as respiratory, urinary, and bloodstream infections, meningitis, sepsis, keratitis, conjunctivitis, and surgical wound infections [13,14]. *S. marcescens* outbreaks in NICUs are responsible for newborn morbidity and mortality [15]. The last European Centre for Disease Prevention and Control (ECDC) report indicates that in 2017, *Serratia* spp. was in 6th and 9th place in European ICU-acquired pneumonia episodes, ICU-acquired bloodstream infection, and ICU-acquired urinary tract infection episodes, respectively [16]. Most clinical *S. marcescens* showed resistance to different classes of antibiotics and, especially, to many extended-spectrum β-lactams [17,18]. In *S. marcescens* multidrug resistance could be intrinsic or plasmid acquired, and the most common mechanism of resistance was that mediated by serine-β-lactamases, such as AmpC, TEM-type, KPC-type, GES-type, and metallo-β-lactamases [18,19,20,21,22,23,24]. Carbapenemases-producing *S. marcescens* strains are more frequently found [25], and they represent a serious risk because this organism is intrinsically resistant to colistin, which is considered the last drug option for carbapenem resistant infections [26]. Simultaneous outbreaks of *S. marcescens* and ESBLs-producing Enterobacterales (i.e., *K. pneumoniae*) have also been described as a situation of greater risk [27,28].

In the present study we have correlate the genetic background of clinical and environmental *S. marcescens* strains during an outbreak occurred in the period February–July 2021 at the NICU of the Pescara Public Hospital (central Italy). The ARGs and virulence factors were examined by whole-genome sequencing. The isolation, partial purification, and kinetic characterization of the SRT-3, AmpC β-lactamases, was also performed.

## 2. Materials and Methods

### 2.1. Clinical Strains

From February to July 2021, eighteen *S. marcescens* clinical strains were isolated from ear, pharyngeal, and rectal swabs of eighteen newborns recovered in NICU of Pescara Public Hospital, central Italy. The NICU is equipped with eight bed stations for the management of the critically ill newborns. The swabs were inoculated in Brain Heart Infusion broth medium (BHI) (Liofilchem, Roseto degli Abruzzi, Italy) and incubated overnight at 37 °C. 100 μL of the overnight cultures were plated on MacConkey agar (Liofilchem s.r.l, Roseto degli Abruzzi, Italy) and incubated at 37 °C for 18 h. One single and pure colony, for each sample, was used to identify *S. marcescens* by Matrix-Assisted Laser Desorption Ionization Time-Of-Flight Mass Spectrometry (MALDI-TOF, MS) (Bruker Daltonics, Billerica, MA, USA).

### 2.2. Environmental Sampling

Environmental samples were collected from numerous surfaces within NICU: doors and door handles, walls, floors, cradles, shelves, benches, hoods, sinks, ventilators, milk collecting devices, medical records, trolleys, stethoscopes, and other personal medical devices. Samples from all environmental surfaces were obtained with a cotton swab wet with a sterile saline solution. The swabs were then inoculated in BHI and then incubated overnight at 37 °C. The procedure was repeated every week for five months. The bacteria identification was performed following the same procedure used for clinical strains, as described in the previous paragraph.

### 2.3. Testing for Antimicrobial Susceptibility

The antimicrobial activity of *S. marcescens* isolates was tested against the routinely clinical antibiotics such as amikacin, amoxicillin/clavulanate, piperacillin/tazobactam, cefepime, cefotaxime, ceftazidime, ciprofloxacin, ertapenem, meropenem, fosfomycin, gentamicin, and colistin using the Phoenix Automated Microbiology System (BD Diagnostic Systems, Sparks, MD, USA). For antimicrobial susceptibility, each bacterial culture was adjusted to a 0.5–0.6 McFarland standard in order to obtain a final inoculum of 5 × 10^5^ CFU/mL. Results for antimicrobial susceptibility were interpreted in accordance with EUCAST guidelines [29].

### 2.4. Whole-Genome Sequencing (WGS)

Total nucleic acid was extracted from *S. marcescens* liquid cultures using a modified protocol of MagMAX Microbiome Ultra Nucleic Acid Isolation kit (Applied Biosystems, ThermoFisher Scientific, Monza, Italy) [30]. Genomic libraries were prepared using Swift 2S Turbo DNA Library kit (Swift Biosciences, Ann Arbor, MI, USA) as previously reported [31]. The WGS was performed on an Illumina MiSeq platform using v3 reagent kits generating 2 × 300 bp paired-end reads (Illumina, San Diego, CA, USA) [32,33]. Raw data from paired-end sequencing were quality checked with the FastQC tool (v.0.11.6) and assembled with Velvet de novo Assembly (v.1.0.0) [34]. Antimicrobial resistance genes were detected using the Comprehensive Antibiotic Resistance Database (CARD) (https://card.mcmaster.ca/analyze/rgi, accessed on 10 February 2022). Genes correlated to the main virulence factors of *S. marcescens* were identified using DFAST annotation and NCBI BLAST (https://blast.ncbi.nlm.nih.gov/Blast.cgi, access on 1 March 2022).

### 2.5. SRT-3 Extraction and β-Lactamase Activity

An overnight culture of *S. marcescens* SM_PE/1 in 1 L of BHI broth was harvested by centrifugation at 2500× *g* (4 °C) for 15 min, then washed twice with 50 mM sodium phosphate buffer pH 6.5. The crude extract was obtained by sonication using a probe of 3 mm in diameter (5 times for 1 min with 2 min off, on ice, at 60 Watt). The cell debris was removed by high-speed centrifugation at 105,000× *g* for 40 min. SRT-3 enzyme was purified in FPLC system using cation exchange Sepharose S FF equilibrated in 50 mM sodium phosphate buffer pH 6.5. The fractions containing SRT-3 were eluted in a linear gradient of NaCl 1 M in the same buffer. The β-lactamase activity was performed following the hydrolysis of some β-lactams (cefazolin, cefotaxime, ceftazidime, cefepime, meropenem) at 25 °C in 25 mM sodium phosphate buffer (pH 7.0). Data were collected with a Perkin-Elmer Lambda 25 spectrophotometer (Perkin-Elmer Italia, Monza, Italy). *K_m_* and *V_max_* were determined under initial-rate conditions using the Hanes-linearization method and the Origin Pro 8.5.1 program to generate Michaelis–Menten curves [35,36].

## 3. Results

### 3.1. Epidemiological Outbreak

*S. marcescens* clinical strains were isolated from 18 newborns (eight boys and ten girls): 15 born preterm (gestational age from 25 to 36 weeks) and 3 born at term. All newborns were hospitalized in the NICU for an average time of 41.4 days. Namely, at birth, all newborns underwent ear, pharyngeal, and rectal swabs, which were found to be positive for *S. marcescens*. Two newborns (gestational age: 25 weeks) were symptomatic and were treated with antibiotics until a negative swab was obtained, with good clinical conditions at the end of the treatment. The antibiotic therapy was meropenem 20 mg/Kg every 12 h for infants younger than 8 days and every 8 h hours for infants older than 8 days. The remaining 16 newborns were asymptomatic, and the swabs were repeated approximately once a week until a negative swab was obtained.

From fifty environmental samples analyzed, one swab, collected from cradle surfaces, was positive for *S. marcescens*. All other medical devices and surfaces analyzed were found to be negative. Immediately after environmental samples microbiological analysis, a sanitation procedure was started, reinforcing all recommended measures such as hand washing with alcohol-based solutions, glove use for assisting and caring newborns, and routine renewal of water and air filters [37]. The last environmental sampling carried out at the beginning of July 2021 yielded negative results.

### 3.2. Antimicrobial Susceptibility

All *S. marcescens* strains, 18 clinical and 1 environmental isolate, were analyzed against a large panel of antibiotics routinely used in the Clinical Microbiology and Virology Unit of Pescara Public Hospital. The *S. marcescens* analyzed showed the same susceptibility profile (Table 1) exhibiting susceptibility to all antibiotics tested, with the exception of amoxicillin/clavulanate association (MIC > 16 mg/L) and colistin (MIC > 8 mg/L).

### 3.3. WGS Analysis

Thus, *S. marcescens* clinical strains of eighteen newborns and one *S. marcescens* isolated from cradle surface were included in the study in order to characterize their WGS. On the basis of bioinformatic analysis, the nineteenth *S. marcescens* showed the genome size ranging from 4.864.007–4.876.696 bp (Table 2). A minor part of the genome was, undoubtedly, loss during library preparation. In all *S. marcescens*, including clinical and environmental strains, no plasmids were identified. However, both clinical and environmental strains showed the same ARGs and virulence factors profile. The *aac(6′)-Ic*, *tetA(41)*, and *bla*_SRT-3_ genes were identified in all strains. Regarding virulence factors, the clinical and environmental *S. marcescens* strains possessed *adeFGH*, *rsmA*, *CRP* elements, and penicillin binding protein-3 gene (PBP3) with D350N amino acid substitution.

### 3.4. Characterization of SRT-3 β-Lactamase

**Figure 1:**
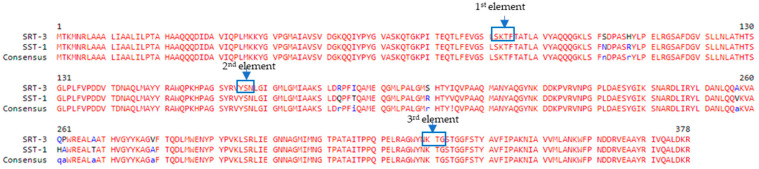
Amino acid sequence alignment of SRT-3 and SST-1 β-lactamases.

The *bla*_SRT-3_ is a β-lactamase gene of 1137 bp encoding for chromosomal class C β-lactamase. The SRT-3 enzyme showed three conserved elements as all serine-β-lactamases. As shown in Figure 1, in SRT-3, the first element, with the active serine, is represented by 64S*LSK73 residues (64S* is the active serine), the second element is the loop 150YSN152, and the third element is 315KTG317. The SRT-3 enzyme exhibited 10 amino acid substitutions with respect to SST-1, which is the constitutive AmpC β-lactamase in *S. marcescens*. The amino acid substitutions are as follows: N86S, R91H, Q168R, T171I, R185S, V242A, H246Q, A247P, T253A, and A264V.

In order to verify the kinetic behavior of the SRT-3, the enzyme was extracted by *S. marcescens* SM_PE/1 and purified by one chromatographic step. The molecular mass and isoelectric point of 41 kDa and 9.2, respectively, were determined for SRT-3. The hydrolysis of SRT-3 was monitored spectrophotometrically against some cephalosporins and one carbapenem. In detail, we have tested cefazolin (2nd generation cephalosporin), cefotaxime and ceftazidime (3rd generation cephalosporins), cefepime (4th generation cephalosporin), and meropenem (carbapenem). Cefazolin was well hydrolyzed by SRT-3 with *V_max_* = 10.5 μmol/s/mg and *K_m_* = 170 μM. On the contrary, cefotaxime, ceftazidime cefepime, and meropenem were not hydrolyzed by SRT-3 enzyme.

## 4. Discussion

*S. marcescens* infections represent a serious problem in hospital settings, in particular in NICUs and ICUs, which are considered the high-risk wards. Our study described an outbreak occurred at NICUs of Pescara Public Hospital (central Italy) involving 18 newborns, two of which were symptomatic. The early etiological diagnosis allowed neonatologists to treat the two symptomatic newborns with a specific antibiotic therapy. Luckily, the outbreak was limited to a small number of cases and none of newborns died. The average time during which the 18 infants were admitted to the NICU was 41.4 days and this probably contributed to an increase of the risk factor for the acquisition of *S. marcescens* infection. It is well known that bacterial colonization in preterm newborns is widespread because their intestinal microbiota has not yet been established [38,39] and their immune system is immature [40,41]. Other risk factors for *S. marcescens* outbreaks are represented by length of stay of newborns in NICU and the broad spectrum administered empirically [42,43].

The extensive collection of environmental samples showed the presence of *S. marcescens* strain only in one cradle, while the other investigated samples yielded negative results. In early 2011, in the same hospital, an outbreak of *S. marcescens* which involved six neonates and the environmental reservoir was represented by two soap dispensers [44]. This is a confirmation that *S. marcescens* outbreak, in the same ward, is a recurring problem. Similar studies have been conducted in other Italian regions [45,46,47,48]. Casolari et al. described two consecutive *S. marcescens* outbreaks in a NICU of a North Italy hospital in a period of 10 years with an overall mortality of 7% [47].

The 19 *S. marcescens* analyzed in the present study showed the typical resistance to colistin and amoxicillin-clavulanate association, but they are susceptible to meropenem which was chosen as preferred antibiotic for therapy. The molecular characterization of both clinical and environmental *S. marcescens* was performed using a next-generation sequencing platform in order to obtain the WGS of each strain. All strains sequenced exhibited the same ARGs and virulence factors profile. The virulence factors *ade-FGH*, *rsmA*, *CRP*, and PBP3, found in our strains, are likely responsible for resistance to various antimicrobials. In particular, the *adeFGH* is one of the three parts which compose the RND efflux pumps (i.e., *AdeABC*, *AdeFGH*, and *AdeIJK*) that contribute to antibiotic resistance in several pathogens [49,50]. The *rsmA* and *CRP* belong to the RND antibiotic efflux pump and it contributed to antibiotic resistance in particular to fluoroquinolone and macrolide resistance [51]. The PBP3 is an essential protein involved in many interactions within the divisome of the bacteria [52] and, as well as other penicillin-binding proteins, represents major targets for β-lactam antibiotics [53]. The substitution D350N in PBP3 has also been found in other bacterial species (i.e., *Haemophilus influenzae*) [54]. Concerning the other resistance genes found in this study, the *aac(6′)-Ic* was identified in 1992 in *S. marcescens* strains and it represents an intrinsic acetyltransferase, which may play a role in primary metabolism of bacterium. The *aac(6′)-Ic* is a chromosomal gene which confers resistance to aminoglycosides (i.e., gentamycin, tobramycin, netilmicin, and amikacin) [55,56]. The *tetA(41)* is a tetracycline efflux pumps protein found in environmental and clinical *S. marcescens* [57,58]. It is most closely related to *tet(39)* protein of *Acinetobacter* [59]. Some ARGs and virulence genes identified in our strains can be commonly found in *S. marcescens* [6].

The SRT-3 enzyme belongs to the SRT AmpC family, which is characteristic of *S. marcescens* clinical isolates [60]. β-lactamases are the commonest cause and the most efficient mechanism of bacterial resistance to β-lactam antimicrobial agents. As confirmed in the last standard numbering scheme [61], three conserved elements have been identified in class C β-lactamases: the first element is the active site with the catalytic serine at position 64 and downstream one helix-turn is located a lysine residue (K67) whose side-chain also points into the active site; the second element 150YSN152 is located on a short loop in the all α domains where it forms one side of the catalytic cavity. The side-chains of the first and third residues point to the active-site cleft; the third element, 315KTG317, is on the innermost strand of the β-sheet (α/β domain) and forms the opposite wall of the catalytic cavity [62]. In the present study, we have characterized the SRT-3 enzyme which showed a narrow spectrum of substrate. Indeed, it was able to hydrolyze only cefazolin, a 2nd generation of cephalosporins. Based on our knowledge, in literature, there are no kinetic data related to SRT-3 enzyme. Similarly, to SST-1, which showed more than 97% of amino acid homology, the SRT-3 is an AmpC enzyme able to hydrolyze only old generation cephalosporins. The *S. marcescens* strains, isolated during the outbreak at NICU of Pescara Public Hospital, did not harbor plasmids or genes encoding for extended-spectrum β-lactamasases or carbapenemases. Thus, all ARGs found in the genome of *S. marcescens* strains were constitutive and not acquired by gene horizontal transfer. We suppose this is due to the fact that, during the same period, no other antibiotic resistant bacteria were identified in NICU.

## 5. Conclusions

In the present study we have described the second *S. marcescens* outbreak that occurred at NICU of Pescara General Hospital in the period 2011–2021. Fortunately, in the recent outbreak only two newborns were symptomatic, and the total number of cases were small. However, ongoing surveillance and infection control programs are necessary to identify the possible infection reservoir in order to adopt, as quickly as possible, the appropriate containment measures.

## Figures and Tables

**Table 1:** Antimicrobial susceptibility of *S. marcescens* clinical and environmental strains.

Antibiotics	*S. marcescens* Clinical Strains (18 Isolates) *S. marcescens* Environmental Strain (1 Isolate)	
	MIC (mg/L)	Interpretation
Amoxicillin/clavulanate	>16	R
Piperacillin/tazobactam	<4	S
Cefepime	<0.12	S
Cefotaxime	<0.12	S
Ceftazidime	<0.12	S
Ertapenem	<0.12	S
Meropenem	<0.12	S
Ciprofloxacin	<0.12	S
Amikacin	<1	S
Gentamicin	<1	S
Fosfomycin	32	S
Colistin	>8	R

**Table 2:** Resistome and virulome analysis of clinical and environmental *S. marcescens*.

Strains (n. Isolates)	Genome Size (bp)	Β-Lactamase Genes	Other ARGs	Virulence Factors
*S. marcescens* (18)	4.864.007–4.876.696	*bla*_SRT-3_	*aac(6′)-Ic* *tetA(41)* *qacG*	*adeFGH* *CRP* *rsmA* *PBP3 (D350N)*
*S. marcescens* (1) cradles	4.874.964	*bla*_SRT-3_	*aac(6′)-Ic* *tetA(41)* *qacG*	*adeFGH* *CRP* *rsmA* *PBP3 (D350N)*

## Data Availability

Not applicable.
